# Toward Unveiling the Mechanisms for Transcriptional Regulation of Proline Biosynthesis in the Plant Cell Response to Biotic and Abiotic Stress Conditions

**DOI:** 10.3389/fpls.2017.00927

**Published:** 2017-06-02

**Authors:** Marco Zarattini, Giuseppe Forlani

**Affiliations:** Laboratory of Plant Physiology and Biochemistry, Department of Life Science and Biotechnology, University of FerraraFerrara, Italy

**Keywords:** proline, P5C synthetase, P5C reductase, osmotic and oxidative stress, transcription factor binding sites, adaptive responses, plant hormones

## Abstract

Proline accumulation occurs in plants following the exposure to a wide array of stress conditions, as well as during numerous physiological and adaptive processes. Increasing evidence also supports the involvement of proline metabolism in the plant response to pathogen attack. This requires that the biosynthetic pathway is triggered by components of numerous and different signal transduction chains. Indeed, several reports recently described activation of genes coding for enzymes of the glutamate pathway by transcription factors (TFs) belonging to various families. Here, we summarize some of these findings with special emphasis on rice, and show the occurrence of a plethora of putative TF binding sites in the promoter of such genes.

## The Genes Coding for the Enzymes of the Glutamate Pathway Are the Putative Target of Many Transcription Factors

Besides its role in protein synthesis, proline has long been known to act as a compatible osmolyte to counteract drought and salinity (Szabados and Savouré, 2010), whereas increasing evidence shows its involvement in the regulation of the cellular redox state (Giberti et al., 2014; Shinde et al., 2016) and in ROS scavenging (Sharma and Dietz, 2006; Liang et al., 2013). Furthermore, proline metabolism seems involved in the induction of the hypersensitive response during the incompatible plant–pathogen interaction (Qamar et al., 2015). Conversely, heat stress does not lead to proline accumulation in *Arabidopsis thaliana*, and induced proline synthesis has further detrimental effect (Lv et al., 2011). Although in many cases increased resistance to water and salt stress has been found in transgenic plants over-accumulating proline (Kavi Kishor et al., 1995; Kumar et al., 2015), its actual role in conferring tolerance is still a matter of debate. It is mainly unclear whether proline accumulation *per se* or the activity of the enzymes controlling its homeostasis is functional to withstand stress conditions (Kavi Kishor and Sreenivasulu, 2014). Therefore, the usefulness and feasibility of proline metabolic engineering for stress tolerance remains an open question (Verslues and Sharma, 2010; Bhaskara et al., 2015).

Two proline biosynthetic pathways have been described in plants. Under high nitrogen availability, ornithine is converted by an ornithine-δ-aminotransferase (OAT) to δ^1^-pyrroline-5-carboxylate (P5C), which is finally reduced by a P5C reductase (P5CR) (da Rocha et al., 2012). However, this route does play a significant role under neither osmotic stress (Funck et al., 2008) nor nitrogen limitation, when P5C is produced from glutamate by a bifunctional P5C synthetase (P5CS) (Hare and Cress, 1997). Convincing evidence for P5CS as the enzyme catalyzing the rate-limiting step in proline synthesis has been described (Kavi Kishor et al., 1995), yet P5CR has been found to be subjected to complex regulation mechanisms at the post-translational level (Giberti et al., 2014; Forlani et al., 2015). Although in most plant species a single gene encodes for P5CR, at least two *P5CS* genes have been usually identified (Fujita et al., 1998; Székely et al., 2008) performing non-redundant functions. In Arabidopsis, *At*P5CS1 is responsible for osmotic stress-induced proline accumulation (Yoshiba et al., 1995; Székely et al., 2008), whereas *At*P5CS2 is essential for seedling growth and embryo maturation (Székely et al., 2008; Funck et al., 2012) and is specifically expressed during incompatible plant–pathogen interactions (Fabro et al., 2004). Conversely, in rice *OsP5CS1* is constitutively expressed, while *OsP5CS2* is primary involved in the response to hyperosmotic stress (Hur et al., 2004).

The regulatory patterns underlying *P5CS1*, *P5CS2*, and *P5CR* gene induction are not fully understood, yet. To date, both abscisic acid (ABA)-dependent and independent signaling pathways are known to lead to osmotic-dependent proline accumulation (Savouré et al., 1997; Ábrahám et al., 2003). In Arabidopsis, ABA-independent *P5CS1* expression has been shown under cold and osmotic stress, while under the same conditions *P5CR* expression did not correlate to proline content (Savouré et al., 1997). A different scenario has been observed in rice, where both *OsP5CS1* and *OsP5CR* are induced by ABA and NaCl treatment (Sripinyowanich et al., 2013).

Eukaryotic gene expression is regulated in a combinatorial manner by transcription factors (TFs) that, binding to different TF binding sites (TFBS) in the promoter region, modulate gene transcription. The analysis of *cis*-regulatory elements (CREs) in a given promoter may therefore represent a useful tool to understand the signal transduction chain underlying the response to a particular stress. Fichman et al. (2015), by using a specific database for Arabidopsis gene sequences^1^, analyzed 1,000 bp upstream the translation start site (TSS) of *AtP5CS1*, *AtP5CS2*, *AtP5CR*, and *AtOAT* genes. In all cases, an impressive number of putative CREs recognized by different TFs classes were found (Fichman et al., 2015). Interestingly, a multiple sequence alignment analysis of the 5′ regulatory region of 48 plant *P5CS1* genes showed a high degree of divergence (supplementary data in Fichman et al., 2015). A higher homogeneity was found for *P5CS2* genes, and the comparison of *A. thaliana* and *A. lyrata* promoters allowed the identification of several CREs known to be recognized by HD-HOX, AP2/EREBP, MYB, WRKY, and bZIP TFs. Concerning *P5CR*, 27 plant sequences were analyzed but, due to their high diversity, no conserved TFBS were identified. Several unique predicted elements were found in *AtP5CR*, including putative bZIP, HD-HOX, MYB and C2C2(Zn)DOF binding sites (Fichman et al., 2015).

**FIGURE 1:**
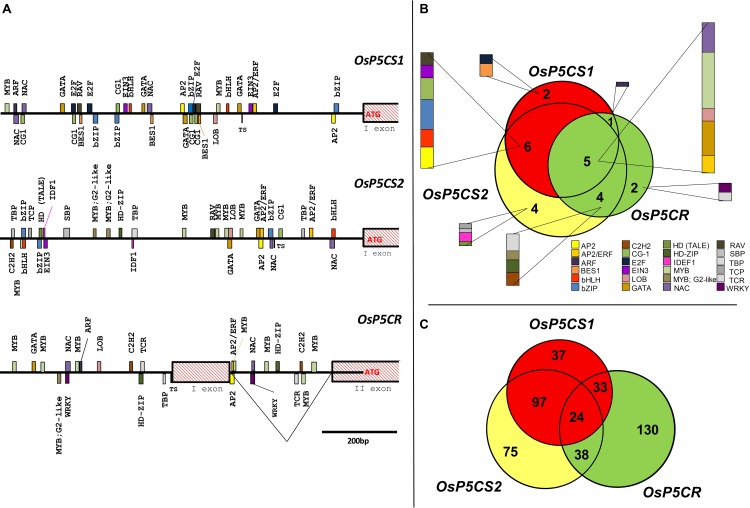
*In silico* analysis of putative *cis*-regulatory elements in *OsP5CS1*, *OsP5CS2*, and *OsP5CR* gene promoters. The genomic context of each gene was identified using the Rice Functional Genomics Database (http://signal.salk.edu/) and the coding sequences were retrieved from NCBI. After identifying the translation start site (TSS), 1000 bp upstream of the ATG were selected and analyzed by using the “promoter analysis” function of PlantPAN 2.0 database (Chang et al., 2008; Chow et al., 2015; http://PlantPan.mbc.nctu.edu.tw). Only putative CREs with a similarity equal or greater than 95% are reported **(A)**. The Venn diagram in **(B)** represents the putative *cis*-regulatory elements in *OsP5CS1*, *OsP5CS2*, and *OsP5CR* gene promoters, whereas the diagram in **(C)** shows the transcription factors which could be able to bind them. The latter have been identified using the “gene search” function of PlantPAN 2.0 database (Chang et al., 2008; Chow et al., 2015; http://PlantPan.mbc.nctu.edu.tw).

Consistent results were found when the presence of putative CREs was investigated in rice (**Figure 1A**). Also in this case dozens of possible TFBS are present, a complete list of which is reported in **Supplementary Table S1**. Interestingly, several differences were evident between *Oryza sativa* and *A. thaliana* genes. Besides some CREs detected in both species that should be recognized by TFs of the MYB, bZIP, and AP2/ERF TF families, a total of 24 different classes of TFs were detected to have a binding site in the promoter of *OsP5CS1*, *OsP5CS2*, and *OsP5CR.* Four of them, namely those belonging to the AP2, GATA, MYB, and NAC TFs families, were present in all genes analyzed. TFBS of the E2F and BES1 families were detected only in the *OsP5CS1* promoter; IDEF1 was unique for the *OsP5CS2* promoter, and TCR and WRKY for *OsP5CR* (**Figure 1B**). If the number of putative TF families identified in rice (24) and Arabidopsis (15) is considered, also taking into account that a more stringent analysis of sequence similarity has been applied in the former (≥95%, this work) than in the latter case (≥50%; Fichman et al., 2015), it seems likely that proline biosynthesis is regulated in rice by a more complex network.

Since more TFs can bind to the same CRE, further analysis allowed to identify numerous TFs putatively able to recognize any of the three promoters (**Supplementary Table S2**), 24 of which could bind to all of them (**Figure 1C**). Furthermore, when the list of TFs identified was subjected to a gene ontology (GO) analysis (**Supplementary Figure S1**), a total of 55 GO terms were found statistically significant, being the top five most enriched terms *metabolic process*, *cellular process*, *primary metabolic process*, *cellular metabolic process*, and *macromolecule metabolic process*. However, though interesting, the results of such *in silico* analyses need to be confirmed by suitable experimental data.

## Regulation of Abiotic Stress-Induced Proline Synthesis by ABA-Dependent Pathway

Abscisic acid-responsive elements (ABREs), belonging to the G-BOX family (ACGTGG/TC) and characterized by an ACGT core sequence, are considered the major *cis*-acting sequences in ABA regulated genes (Shinozaki and Yamaguchi-Shinozaki, 2007). In order to drive ABA-induced expression, the presence of at least an ABRE copy associated with a coupling element (CE) is required. In rice, two sequences containing a G-box element were found only in the promoter of *OsP5CS1*, 73 and 481 bp upstream of the TSS, whereas a single sequence containing the CCACC core sequence of CE1 is present 300 bp upstream of the translation starting site. A large part of the bZIP family is able to bind a sequence containing an ACGT core [class A in Arabidopsis, also referred to as ABRE binding factor (ABF) and ABA-responsive element binding protein (AREB)]. In Arabidopsis and rice 75 and 92 bZIPs proteins have been identified, respectively. Numerous transgenic lines ectopically overexpressing bZIP proteins have been shown to be more sensitive to ABA treatment and more resistant to drought and salinity (Tang et al., 2012; Zong W. et al., 2016). Recently, Xu et al. (2016) reported that transgenic Arabidopsis plants carrying the soybean *GmbZIP110* gene were capable of accumulating significant amounts of proline even if *AtP5CS1* transcription was not apparently induced. Likewise, transgenic Arabidopsis plants overexpressing the wheat *TabZIP60* contained significantly higher amounts of proline (Zhang et al., 2015). Even if further experimental studies are required, these data strongly support the possibility that the signaling pathway mediated by TFs of the bZIP family is involved in the regulation of proline biosynthesis.

Another group of G-BOX (and E-BOX) binding factors is represented by the large bHLH family, 162 and 111 members of which have been identified in Arabidopsis and rice, respectively. Similarly to bZIP, the overexpression of specific bHLH proteins led to increased proline levels, resulting in turn into higher tolerance to osmotic (Liu et al., 2014, 2015) and cold (Jin et al., 2016) stress. Recently *At*bHLH112 was found able to bind also the GCG-BOX and act as a transcriptional activator (Liu et al., 2015). The overexpression of this protein induced increased proline accumulation, as well as the induction of both *AtP5CS* isoforms following ABA, NaCl, and mannitol treatment. Consistently, several GCG-box motifs were found in both genes supporting a role for this TF in the regulation of proline biosynthesis (Liu et al., 2015). Indeed, two G-BOX motifs, specific for bHLH, are present also in both 1 Kbp *OsP5CS* promoters (**Figure 1A** and **Supplementary Table S1**).

## Regulation of Abiotic Stress-Induced Proline Synthesis by ABA-Independent Pathway

Dehydration-responsive elements (DRE), DRE-related motifs such as C-repeats (CRT) and low-temperature-responsive elements are considered to be the major CREs responsible for ABA-independent stress-responsive gene induction. Unlike ABRE, a single DRE copy is sufficient to drive gene expression. TFs belonging to the ERF/AP2 family able to bind DRE/CRT elements were termed DREB1/CBF and DREB2. In particular, the DREB1-type genes are involved in cold-responsive pathways, whereas DREB2-type genes play a role in osmotic-responsive pathways. Several studies demonstrated that the overexpression of either DREB1 or DREB2 genes improved plant tolerance to drought, salt and freezing (e.g., Lata and Prasad, 2011). In this frame, some evidence supported their activity as *P5CS* transcriptional regulators (Zhang et al., 2013, 2016). In particular, soybean plants overexpressing *Os*DREB2A showed higher *GmP5CS* expression, despite the absence of any DRE sequence in *GmP5CS* promoter (Zhang et al., 2013). However, some DREB proteins are also able to bind to a GCC-box (Franco-Zorrilla et al., 2014), and in fact a GCC *cis*-acting element was found in *GmP5CS* promoter. Moreover, the overexpression in rice of *Aa*DREB1 protein from the cold-tolerant plant *Adonis amurensis* caused a two-fold increase of free proline under both permissive and cold stress conditions (Zong J.M. et al., 2016). Concerning rice, only a partially identical DRE sequence (tCCGAC) is evident 421 bp upstream of the *OsP5CR* TSS, and a sequence matching the DRE core ACCGAC is found 72 bp downstream of the ATG start codon of *OsP5CS2*. This notwithstanding, and consistently with the above-mentioned results in soybean, two GCC-like elements are present in the promoter of *OsP5CS1* (at -300 bp and -505 bp).

**FIGURE 2:**
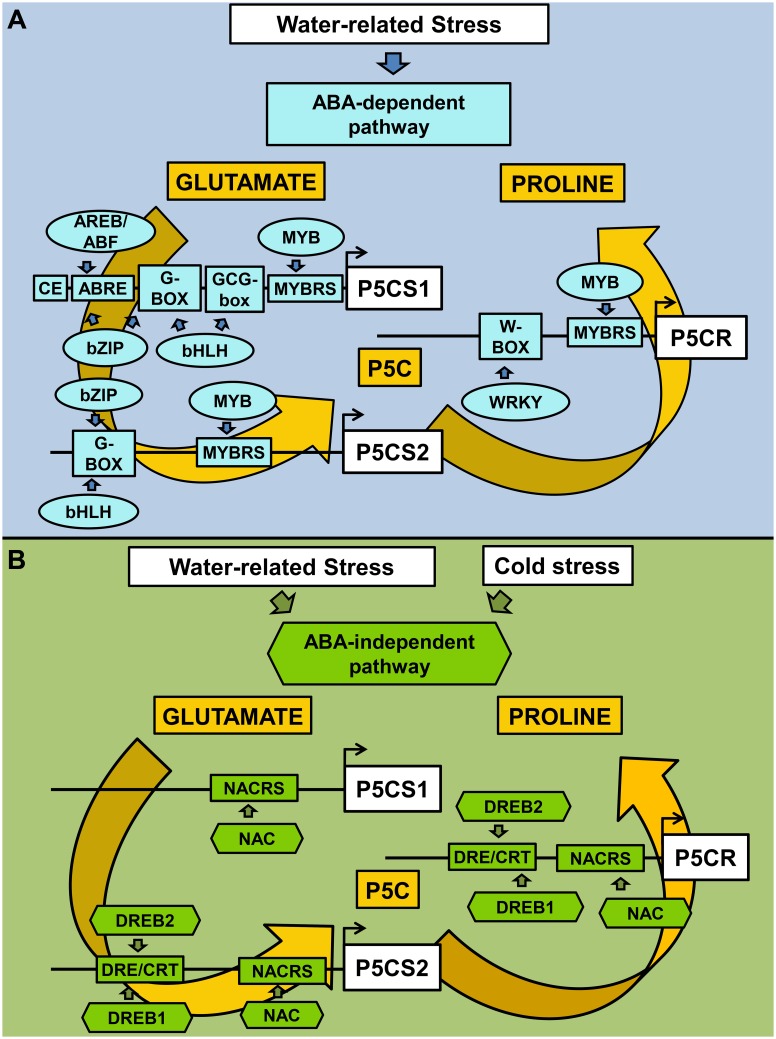
Transcriptional regulatory network of proline biosynthesis as mediated by ABA-dependent and ABA-independent pathways. Water and cold stresses activate abscisic acid-dependent and/or –independent signaling pathways, which in turn modulate specific classes of TFs. CREs and TFs involved in ABA-dependent activation of proline biosynthesis are reported in sky blue **(A)**, while those involved in ABA-independent pathway are reported in green **(B)**. ABRE, ABA responsive elements; CE, coupling element; DRE/CRT, dehydration-responsive elements/C-repeat; MYBRS, MYB recognition sequences; NACRS, NAC recognition sequences. Proline accumulation has been reported to occur in response to a variety of stress conditions, under which its possible role may vary from that of a compatible osmolyte lowering the intracellular water potential to avoid water withdrawal from the apoplast (as in the case of drought and salinity, but also under freezing cold), to a protectant for proteins (at high ion concentration) and membranes (to cope with excessive salt or freezing), or an antioxidant (in response to a direct oxidative stress as that caused by heavy metals, or to mitigate oxidative conditions triggered by other stress types). As a function of these different roles, different cytoplasmic levels of proline are most likely needed. The presence of many CREs recognized by various TFs may allow a fine regulation of gene transcription and facilitate the attainment of a certain homeostatic level, as required to deal with a given stressor. Moreover, at the cellular level the effect of different stresses is virtually the same. For instance, cell dehydration occurs as a consequence of drought, excess salt in the soil, or ice crystal formation in the apoplast. The presence of apparently redundant regulatory networks, as the ABA-dependent and ABA-independent pathways, may therefore be functional to distinguish among these different stress conditions. However, a *cross-talk* between them is essential to ensure the attainment of a suitable response. Despite the importance of this aspect for a proper understanding of the plant cell reaction to stress, to date our knowledge of how the two signaling pathways regulate each other is still limited, or restricted to partial aspects (Roychoudhury et al., 2013; Yoshida et al., 2014). The flux through one pathway may affect the other, and they might act in an additive or negatively regulatory way, or might compete for a target. Shared elements are expected to work as *nodes*, allowing the cross-talk. Since recent data suggest that high intracellular proline levels may induce in turn the catabolic pathway influencing mitochondrial respiration and reactive oxygen species generation (Ben Rejeb et al., 2014; Cabassa-Hourton et al., 2016), proline synthesis could represent one of these nodes. Further work is required to shed light on this possibility.

Another class of plant-specific TFs, namely the NAC (NAM, ATAF, and CUC) proteins, is involved in the ABA-independent pathway under stress. NAC proteins are a wide family, with almost 110 members in Arabidopsis and 151 members in rice. The DNA binding sequence is heterogeneous, but a CACG core-DNA binding motif has been identified in different drought-inducible promoters. In several cases, the overexpression of NAC genes resulted in increased drought/salt tolerance and higher free proline levels (Liu et al., 2013; Hong et al., 2016). However, most members of the NAC family have not been characterized, yet, and suitable functional studies are still required (Shao et al., 2015). The CACG NAC-core motif is present in *OsP5CS2* and *OsP5CR* promoters, but several other NAC binding motifs have been identified in all three promoters analyzed (**Figure 1A** and **Supplementary Table S1**), suggesting that this TF family might regulate proline accumulation under both stressful and permissive conditions. On the whole, the transcriptional regulatory network of proline biosynthesis as mediated by ABA-dependent and ABA-independent pathways is shown in **Figure 2**.

## Regulation Mediated by Apetala2/Ethylene Responsive Factors (AP2/ERF): A Node Between Abiotic and Biotic Signaling Pathway?

The Apetala2/Ethylene Responsive Factors (AP2/ERF) are a superfamily of TFs characterized by the AP2 DNA binding domain. Based on the number of repeated AP2 domains, three families have been defined: ERF, AP2, and RAV. The ERF family is further divided in two sub-families with different DNA binding specificity, ERF and CBF/DREB. The latter is associated with the plant response to abiotic stress, whereas the former (binding the GCC-box) plays a role in biotic and abiotic stress responses, as well as in response to the treatment with jasmonic acid, ethylene, wounding and during development (Dey and Volt, 2015). The wheat ERF1 gene (*TaPIE1*) has been shown to confer resistance to both the necrotrophic pathogen *Rhizoctonia cerealis* and freezing. Promoter analysis and binding affinity assay showed that *TaPIE1* is able to bind a GCC-box within the promoter of *TaP5CR*, thereby promoting its expression (Zhu et al., 2014). Several other studies also showed that the overexpression of ERF members is positively correlated with increased osmotic stress tolerance due to proline accumulation (Rong et al., 2014; Wang et al., 2015; Yao et al., 2015). Moreover, both P5CS transcripts were significantly more abundant in *Jatropha curcas* overexpressing ERF2 than in wild-type plants (Wang et al., 2015). On the other hand, some ERF genes negatively regulate stress tolerance. Recently, the *Bp*ERF11 from *Betula platyphylla* was found to specifically bind both GCC-box and DRE sequences. Interestingly, its overexpression resulted in decreased osmotic stress tolerance in connection with both downregulation of the proline biosynthetic genes *BpP5CS1* and *BpP5CS2* and upregulation of the proline catabolic genes *BpProDH* and *BpP5CDH* (Zhang et al., 2016). Two GCC-box sites were found in *OsP5CS1* promoter. However, due to the complexity of the roles of ERF proteins, no conclusion can be drawn on their possible significance.

## Other Transcription Factors Mediating Proline Biosynthesis: WRKY, CaMTA, and MYB

The WRKY superfamily of TFs targeting the W-box (TTGACC/T) plays a key role in plant defense signaling, yet additional roles in abiotic stress response are emerging (Banerjee and Roychoudhury, 2015). Some studies showed that WRKY members may have a regulatory role on proline metabolism. Wheat *TaWRKY10* overexpressed in tobacco conferred tolerance to salt and drought due to increased intracellular proline levels (Wang et al., 2013). Transgenic rice overexpressing *AtWRKY57* showed increased expression of *OsP5CS1* under hyperosmotic conditions (Jiang et al., 2016). No experimental data have been reported to date on P5CR, but two W-box sites were found in the promoter of *OsP5CR* (**Figure 1A**).

As a major Ca^2+^ sensor protein, calmodulin (CaM) plays a pivotal role in biotic and abiotic stress signaling. A sequence-specific DNA-binding domain is conserved among calmodulin-binding transcription activators (CaMTAs) proteins, and the DNA *cis*-element that binds to CaMTA was identified as (G/A/C)CGCG(C/G/T). In *A. thaliana* a Ca^2+^-dependent CaM-binding protein was found to interact with *At*MYB2 that in turn is able to upregulate several genes among which *AtP5CS1*, enhancing salt tolerance (Yoo et al., 2005). Moreover, microarray data showed that under drought, salt, and cold stress CaMTA1 upregulates both *AtP5CS1* and *AtP5CS2* gene expression in roots, but not in leaves (Supplementary data in Pandey et al., 2013). A set of TFBS for CaMTAs was in fact found in the promoter of rice *OsP5CS1* and *OsP5CS2* genes (**Figure 1A**, namely CG-1).

Lastly, MYB factors represent one of the largest TF families in plants. Based on the presence of one, two, or three repeats in their DNA-binding domain, they are classified into three subfamilies: MYB-related group, MYBR2R3, and MYBR1R2R3, respectively. Members of the MYB family have been found to be involved in the plant response to various abiotic stresses including salt, drought, cold, and excessive light (Li et al., 2015). Several MYB recognition sequences have been identified in the promoter of proline biosynthesis genes both in rice (this work) and Arabidopsis (Fichman et al., 2015). In several studies a high correlation was found between expression of members of the MYB family and proline levels (Shukla et al., 2015; Li et al., 2016). Overexpression of MYB2 induced proline accumulation in Arabidopsis (Yoo et al., 2005), wheat (Mao et al., 2011), and rice (Yang et al., 2012). In the last case a direct induction of proline biosynthesis was proved. Similarly, *Os*MYB48-1 overexpressing rice plants had higher expression levels of both *OsP5CS1* and *OsP5CS2*, and accumulated higher amounts of proline under drought (Xiong et al., 2014).

## Concluding Remarks

Proline metabolism plays a crucial role in the plant response to various abiotic and biotic stress conditions. As such, its synthesis needs to be finely regulated by multiple signaling pathways. Consistently, *in silico* analysis of gene promoter regions allowed the detection of a plethora of putative TFBS in any of the three genes coding for the enzymes responsible of the conversion of glutamate into proline. Considerable evidence was previously obtained confirming that proline synthesis under osmotic stress is driven by both ABA-dependent and ABA-independent signaling. Emerging data suggest that the expression of proline biosynthetic genes is regulated by many TFs that are related to almost all plant hormones.

However, supporting experimental data are needed to substantiate this possibility, and shed light on the whole network regulating proline production under physiological –either stressful or non-stressful– conditions. Recently, several *in vivo* and *in vitro* approaches have been used to study transcriptional regulatory networks governed by specific TFs. Among these, chromatin immunoprecipitation, followed by microarray or sequencing, and yeast one hybrid assay are considered as the most promising (Franco-Zorrilla and Solano, 2017). We are currently trying to use the promoter trapping method (Jiang et al., 2006), in which a given promoter region putatively binding a TF is amplified by PCR with two (GT)_5_ tails at each 3′ end. Following incubation of the amplified fragment with nuclear extracts prepared at increasing time after the exposure to stress conditions, the protein-promoter complex possibly obtained is purified by affinity chromatography on a (AC)_5_-Sepharose column. This approach, once optimized, should allow us to identify some TFs that are truly able to bind promoters of the proline biosynthesis genes. Once a putative signaling pathway component has been identified in this way, the effect of null mutations on proline homeostasis under stress, as well as the results of ectopic expression studies, may be used to define its exact role. The use of these techniques is expected in the near future to help understand the molecular switches controlling proline biosynthesis, and therefore increase our knowledge of mechanisms underlying crop stress tolerance.

## Author Contributions

Both authors have made substantial, direct, and intellectual contribution to the work, and approved it for publication.

## Conflict of Interest Statement

The authors declare that the research was conducted in the absence of any commercial or financial relationships that could be construed as a potential conflict of interest. The reviewer CJDO and handling Editor declared their shared affiliation, and the handling Editor states that the process nevertheless met the standards of a fair and objective review.

## Footnotes

1 www.athamap.de

## Supplementary Material

**FIGURE S1** | Gene ontology (GO) enrichment analysis of TF genes putatively interacting with the promoter of *OsP5CS1*, *OsP5CS2* and *OsP5CR* genes. Enriched GO terms with false discovery rates (FDR < 0.05) from AgriGO analysis (Du et al., 2010) were submitted to the REVIGO program (Supek et al., 2011). GO categories are represented by circles and are visualized by clustering based on semantic similarities to other GO terms. Circle size is proportional to the frequency of each GO term, whereas color indicates the log10 *p*-value for the enrichment analysis.

**TABLE S1** | Complete list of putative *cis*-regulatory elements in the promoter of *OsP5CS1*, *OsP5CS2*, and *OsP5CR* genes. CREs were detected by using the “promoter analysis” function of PlantPAN 2.0 database (Chang et al., 2008; Chow et al., 2015; http://PlantPan.mbc.nctu.edu.tw), and are listed with regard to the position, strand, similarity, and related TF family.

**TABLE S2** | List of putative TF genes for which at least a TFBS has been found in the promoter of *OsP5CS1*, *OsP5CS2*, and *OsP5CR* genes. TFs have been identified by using the “gene search” function of PlantPAN 2.0 database (Chang et al., 2008; Chow et al., 2015; http://PlantPan.mbc.nctu.edu.tw).
