# Supplementary material for: Toward Unveiling the Mechanisms for Transcriptional Regulation of Proline Biosynthesis in the Plant Cell Response to Biotic and Abiotic Stress Conditions
Source: Front Plant Sci. 2017 Jun 2;8:927. doi: 10.3389/fpls.2017.00927 (PMC5454058; doi:10.3389/fpls.2017.00927)
Supplement: Supplementary file 1 [file Image_1.PDF]

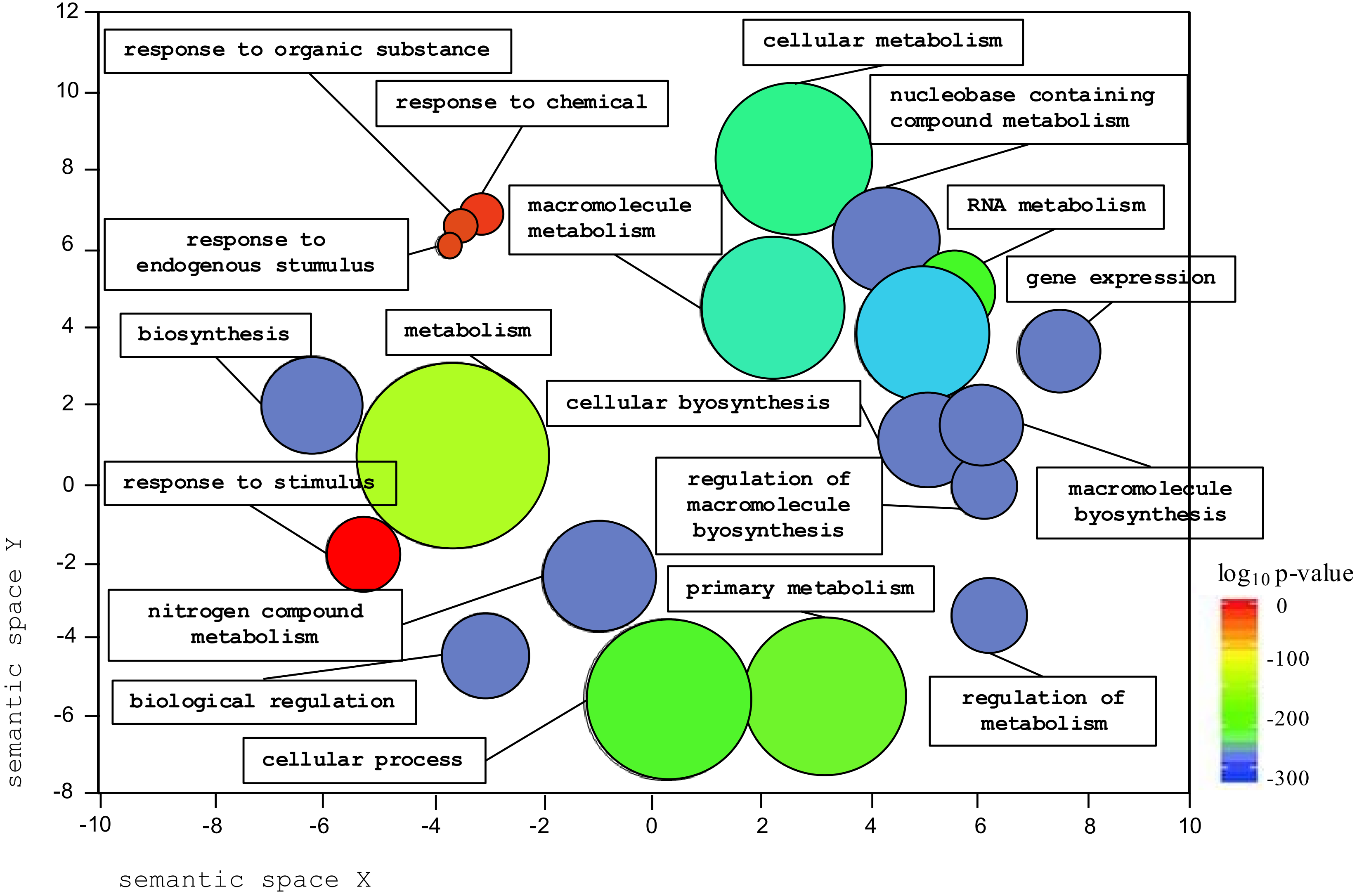

Figure S1 │ Gene ontology (GO) enrichment analysis of TF genes putatively interacting with the promoter of *OsP5CS1*, *OsP5CS2* and *OsP5CR* genes. Enriched GO terms with false discovery rates (FDR < 0.05) from AgriGO analysis (Du et al., 2010) were submitted to the REVIGO program (Supek et al., 2011). GO categories are represented by circles and are visualized by clustering based on semantic similarities to other GO terms. Circle size is proportional to the frequency of each GO term, whereas color indicates the log10 p-value for the enrichment analysis.

Du, Z., Zhou, X., Ling, Y., Zhang, Z., and Su, Z. (2010). agriGO: a GO analysis toolkit for the agricultural community. *Nucleic Acids Res.* 1; 38: W64–W70. doi: 10.1093/nar/gkq310

Supek, F., Bošnjak, M., Škunca, N., and Šmuc, T. (2011). REVIGO summarizes and visualizes long lists of gene ontology terms. *PLOS ONE* 6:e21800. doi:10.1371/journal.pone.0021800
